# Clinical Severity of β-thalassaemia/Hb E Disease Is Associated with Differential Activities of the Calpain-Calpastatin Proteolytic System

**DOI:** 10.1371/journal.pone.0037133

**Published:** 2012-05-16

**Authors:** Suriyan Sukati, Saovaros Svasti, Roberto Stifanese, Monica Averna, Nantika Panutdaporn, Tipparat Penglong, Edon Melloni, Suthat Fucharoen, Gerd Katzenmeier

**Affiliations:** 1 Institute of Molecular Biosciences, Mahidol University, Salaya Campus, Nakorn Pathom, Thailand; 2 Department of Biochemistry, Faculty of Science, Mahidol University, Bangkok, Thailand; 3 Biochemistry Section, Department of Experimental Medicine (DI.ME.S.), and Centre of Excellence for Biomedical Research (C.E.B.R), University of Genoa, Genoa, Italy; University of Western Ontario, Canada

## Abstract

Earlier observations in the literature suggest that proteolytic degradation of excess unmatched α-globin chains reduces their accumulation and precipitation in β-thalassaemia erythroid precursor cells and have linked this proteolytic degradation to the activity of calpain protease. The aim of this study was to correlate the activity of calpain and its inhibitor, calpastatin, with different degrees of disease severity in β-thalassaemia. CD34^+^ cells were enriched from peripheral blood of healthy individuals (control group) and patients with mild and severe clinical presentations of β^0^-thalassaemia/Hb E disease. By *ex vivo* cultivation promoting erythroid cell differentiation for 7 days, proerythroblasts, were employed for the functional characterization of the calpain-calpastatin proteolytic system. In comparison to the control group, enzymatic activity and protein amounts of μ-calpain were found to be more than 3-fold increased in proerythroblasts from patients with mild clinical symptoms, whereas no significant difference was observed in patients with severe clinical symptoms. Furthermore, a 1.6-fold decrease of calpastatin activity and 3.2-fold accumulation of a 34 kDa calpain-mediated degradation product of calpastatin were observed in patients with mild clinical symptoms. The increased activity of calpain may be involved in the removal of excess α-globin chains contributing to a lower degree of disease severity in patients with mild clinical symptoms.

## Introduction

Thalassaemia is an inherited disorder occurring with high prevalence in Southeast Asia. In Thailand, α-thalassaemias attain frequencies of up to 30% and β-thalassaemias vary from 3–9% in different populations [1]. In severe cases of β-thalassaemia, the accumulation and subsequent precipitation of excess unpaired α-haemoglobin chains in red cell precursors causes a number of pathological symptoms such as ineffective erythropoiesis, anaemia and haemolysis which ultimately can result in skeletal abnormalities and eventually organ damage such as cardiac failure [2]. Mutations in the β-globin gene can either lead to impaired synthesis (β^+^-thalassaemia) or complete absence (β^0^-thalassaemia) of β-globin chains [3] and polymorphisms in the β-globin cluster leading to the disease phenotype have been extensively characterized [4], [5]. Hb E, one of the most common haemoglobin variants with frequencies of up to 50% in the border region of Laos, Cambodia and Thailand, displays a G→A substitution in codon 26 of the β-globin gene (β^E^). It is probably the most common β-thalassaemia allele worldwide. Compound heterozygotes with a coinherited deficiency in a second β-thalassaemia allele leading to β^0^-thalassaemia/Hb E disease, can demonstrate a highly variable presentation of disease symptoms despite having apparently identical genotypes. The remarkable variation of disease severity can range from nearly asymptomatic (‘mild’ form) to transfusion-dependent anaemia (‘severe’ form).

The major factor involved in the pathophysiology of β-thalassaemia is probably the high amount and precipitation of excess α-globin chains which leads to subsequent oxidative damage of developing red cells. Among other inherent factors that were proposed as possible modulators of disease severity were elevated Hb F production, erythropoiesis and proteolysis in the erythrocyte [6]. The latter was suspected to affect the severity of thalassaemia by a reduction of the amount of excess α-globin chains and thereby ameliorating the pathological effects of globin chain imbalance to the cell [6]. Earlier observations in the literature have demonstrated that the proteolytic processes involved in haemoglobin breakdown entail pathways which are dependent on ubiquitin, ATP and intracellular Ca^2+^-ions [7], [8].

Previous studies have used mature erythrocytes for the analysis of proteolytic globin degradation [9], [10], however, several reports have suggested that globin breakdown is accelerated in the bone marrow when compared to peripheral blood reticulocytes [11], [12] and that the degree of globin degradation in erythroid precursor cells is reflective of an ineffective erythropoiesis in severe cases of β-thalassaemia [13]. Moreover, a number of reports published later indicated that the activity of calpain and calpain activator (CA) progressively decreases during the maturation and differentiation of erythroid precursor cells [14], [15]. We have therefore decided to cultivate enriched CD34^+^ precursor cells to the proerythroblast stage and used for the analysis of calpain and calpastatin activity.

With the aim to seek further confirmatory evidence for a role of the calpain-calpastatin proteolytic system as modulator of disease phenotypes in β^0^-thalassaemia/Hb E patients, we have undertaken a comparative biochemical analysis of calpain activity in samples from patients with mild and severe symptoms. The findings presented in this study support the view that activity of calpain and its inhibitor, calpastatin, may be of functional significance for the presentation of disease symptoms in β-thalassaemia.

## Materials and Methods

### 2.1 Ethics statement

The research described herein was carried out in full compliance with the Helsinki declaration. Study design and informed consent form for patients were approved by the Committee on Human Rights Related to Human Experimentation of Mahidol University, Nakorn Pathom, 73170 Thailand (reference number MU 2006-139,. File S1). Patients agreed to participate in the study by signing a written consent form translated into their native (Thai) language (File S2). No animals were utilized in this study to produce recombinant calpastatin from rat brain as described in an earlier publication [16].

### 2.2 Subjects

Thai/Chinese β^0^-thalassaemia/Hb E patients were categorized into groups with mild and severe clinical symptoms according to a scoring system for the severity of β-thalassaemia/Hb E disease [17]. Healthy volunteers were identified by using haemoglobin typing (HPLC β-globin variant short program, Bio-Rad Laboratories, Hercules, CA, USA) and were recruited as control group. Multiplex polymerase chain reaction was used to screen for mutations in α-thalassaemia genes [18]. In order to reduce possible genetic background effects, only β^0^-thalassaemia/Hb E disease patients carrying mutations in β^codon 41/42; −TTCT^ and β^E^ were selected for this study. Patients with co-inherited α-thalassemia were excluded from the study and patients with mild and severe symptoms had no significant differences in their Hb F amounts (37.57±1.78% and 37.56±2.72%, respectively). Patients did not receive blood transfusions for at least one month and medication for at least 2 weeks prior to blood sample collection. Subjects had no other health issues and were free of infections at the time of sample collection.

### 2.3 Purification of CD34^+^pheripheral blood mononuclear cells (PBMC)

Peripheral blood (30 ml from β^0^-thalassaemia/Hb E patients and 50 ml from healthy controls) of peripheral venous blood were collected in heparin (0.2 mg ml^−1^ blood) and PBMC were obtained by centrifugation at 800×g for 20 min at room temperature on Lymphoprep™ (AXIS-SHIELD PoC AS, Oslo, Norway). CD34^+^ cells were selected from PBMC using anti-CD34 magnetic microbeads and magnetic activated cell sorting (MACS) separation columns (both from Miltenyi Biotech, Auburn, CA, USA) according to the manufacturer's protocol.

### 2.4 Erythroid precursor cell culture

CD 34^+^cells were grown under standard culture conditions [19]. Briefly, isolated peripheral CD34^+^ cells were cultured in Iscove's Modified Dulbecco's Media (IMDM; GIBCO BRL, Grand Island, NY, USA) containing 1% deionized bovine serum albumin (Stem Cell Technologies Inc. Vancouver, BC, Canada), 15% heat inactivated foetal calf serum (FCS; GIBCO BRL), 15% human serum type AB from healthy male donors, 100 U ml^−1^ penicillin, and 100 U ml^−1^ streptomycin (GIBCO BRL) with the following recombinant cytokines: 2 U ml^−1^ recombinant human erythropoietin (rhEPO; EPREX, Brussels, Belgium), 20 ng ml^−1^ recombinant human stem cell factor (rhSCF; Promokine, Heidelberg, Germany), and 10 ng ml^−1^ recombinant human interleukin-3 (rhIL-3; Promokine), then incubated at 37°C under 5% CO_2_. On day 3, cells were centrifuged at 400×g for 7 min at room temperature and the collected cells were incubated under identical conditions without rhIL-3. Cells were cultured for 7 days and used for further experiments. Purity of erythroid cells was determined by staining cells with erythroid specific-cell surface makers, allophycocyanin (APC)-conjugated monoclonal mouse anti-human glycophorin A (CD235a) and phycoerythrin (PE)-conjugated monoclonal mouse anti-human transferrin receptor (CD71) (BD Bioscience, Pharmingen, San Diego, CA, USA). Stained cells were analyzed by a flow cytometer (FACSCalibur, BD Biosciences, San Jose, CA, USA). Signal areas (R1–R3) were gated for the detection of glycophorin A (CD235a) and transferrin receptor (CD71). Cell morphology was evaluated by cytospin preparation (Cytofuge 2, Statspin, Inc., Norwood, MA, USA) for 5 min at 1,000 rpm following staining with Wright-Giemsa dye. Cells were analyzed by using differential counting with light microscopy (magnification 1000-fold) (Olympus CH-2, Olympus Optical Co. Ltd, Tokyo, Japan).

### 2.5 Preparation of recombinant rat brain calpastatin RNCAST300

Recombinant rat brain calpastatin RNCAST300, composed of exon 8 and a single inhibitory unit (exons 9–12), has been prepared, purified and assayed as described previously [16]. In order to completely inhibit calpain activity, 10 nM (1.1 µg ml^−1^) RNCAST300 was added to the cell lysates.

### 2.6 Determination of calpain activity

5×10^6^ cells were lysed in 500 µl of ice-cold hypotonic medium 50 mM sodium borate buffer, pH 7.5, containing 0.5 mM 2- mercaptoethanol (buffer A) and 5 mM EDTA by three cycles of freezing and thawing in liquid nitrogen. The particulate material was separated by centrifugation at 100,000×g for 10 min at 4°C and discarded. Cytosolic fractions were collected and utilized for assay of calpain activity with the fluorogenic peptide substrate Ac-LLY-afc (BioVision Inc., Mountain View, CA, USA) and of 1 mM Ca^2+^ over the EDTA present in the samples. As controls, the same incubation mixtures were carried out in the presence of 5 mM EDTA. The dependency of the detected proteolytic activity from calpain was established by adding to the assay incubation mixtures at a final concentration of 10 µM the irreversible calpain inhibitor Z-LLY-fmk [20], [21] or 1.1 µg ml^−1^ recombinant calpastatin form RNCAST300 [16]. Fluorescence was detected on a spectrofluorometer (Wallac 1420 Victor 2, Perkin Elmer, Wellesley, MA, USA) at excitation wavelength λ = 400 nm and emission wavelength λ = 505 nm. Calpain activity is calculated and reported throughout the text on the basis of the difference between the fluorescence values detected from the complete incubation mixtures and the fluorescence values obtained with incubation mixtures containing calpain inhibitors or EDTA. Gross variations of the metabolism in cultured erythroid precursor cells were excluded by determination of activity of glycerol aldehyde-3-phosphate dehydrogenase (GAPDH) using the KDalert™ GAPDH Assay Kit (Ambion Inc., Austin, Texas, USA) according to the manufacturer's instructions. All assays were performed in duplicate and numerical values are presented as mean plus or minus standard error.

### 2.7 Western blot analysis

10^6^ cells were lysed with 35 µl ice-cold lysis buffer (50 mM Tris-HCl, pH 7.5, 15 mM NaCl, 1% triton X-100, 1 mM EDTA, 1 mM EGTA, 10 mM sodium β-glycerophosphate, 5 mM sodium pyrophosphate, and protease inhibitor cocktail, Sigma-Aldrich). Protein samples (10 µg) were run on 8% SDS-PAGE and subsequently transferred to polyvinyl difluoride (PVDF) membranes. Membranes were probed with mouse monoclonal anti-μ-calpain (large subunit) at 1∶1,000 dilution (Chemicon International Inc., San Diego, CA, USA) and a monoclonal mouse anti-GAPDH antiserum (Santa Cruz Biotechnology Inc., Santa Cruz, CA, USA) at 1∶7,500 dilution. Alternatively, protein samples (25 µg) were run on 6%–10% gradient SDS-PAGE and subsequently transferred to PVDF membranes. Membranes were probed with mouse monoclonal α-spectrin II antibody at 1∶1,000 dilution (Chemicon) and anti-GAPDH (Santa Cruz Biotechnology Inc.) at 1∶10,000 dilution. Bands were visualized using horseradish peroxidase conjugated anti-mouse IgG (GE Healthcare UK limited, Buckinghamshire, England, dilution 1∶5,000) with the ECL plus Western blotting detection system (GE Healthcare). Band intensities of proteins were analyzed by densitometric scanning of the membrane and quantified using the ImageJ 1.41 program (National Institutes of Health, Bethesda, USA).

### 2.8 Determination of calpastatin activity

Calpastatin activity in cell lysates was analyzed by procedures described previously in the literature [22]. 5×10^7^ erythroid precursors cultured from 4 healthy controls, 5 patients with mild and 5 patients with severe clinical symptoms, respectively, were lysed in 625 µl of ice-cold buffer A containing 1 mM EDTA as previously described. Cytosolic fractions, prepared as previously described, were collected, heated for 3 min at 100°C, and precipitated proteins were removed by centrifugation at 100,000×g for 10 min at 4°C. The supernatant was loaded onto a diethylaminoethyl cellulose resin (DE53) anion exchange chromatography column (1×5 cm) (Whatman, Hillsboro, OR, USA) equilibrated with buffer A containing 0.1 mM EDTA. Proteins were eluted with a linear gradient from 0 to 0.5 M NaCl at a flow rate of 1 ml min^−1^ and fractions of 1 ml were collected. Calpastatin activity was measured using purified human erythrocyte calpain prepared as reported in [22] and human acid-denatured globin as a substrate as described earlier [23].

### 2.9 Identification of calpastatin species by SDS-polyacrylamide gel electrophoresis

Calpastatin fragments were analysed as described previously [24]. 6×10^7^ erythroid precursor cells cultured from 4 controls, 5 patients with mild and 5 patients with severe clinical symptoms, respectively, were prepared and lysed as described above. Supernatants were concentrated to 50 µl by ultrafiltration on an Amicon centrifugation tube (MW cut off 10 kDa) and proteins were separated on large format (16×16 cm) 12% SDS-polyacrylamide gels. Gels were washed 6 times for 10 min each with buffer A containing 0.1 mM EDTA and 20% methanol, to remove SDS and subsequently were washed 3 times each for 10 min with buffer A containing 0.1 mM EDTA to remove methanol. Gels were cut into 3 mm thick slices and proteins were extracted from each slice by incubation with 600 µl extraction buffer (buffer A containing 0.1 mM EDTA) in continuous and gently shaking at room temperature for 48 hr.

### 2.10 Statistical analysis

Results are expressed as the mean plus standard error and the mean plus or minus standard error. Experimental groups were compared using the nonparametric test, with a *p* value of less than 0.05 indicating statistical significance.

## Results

### 3.1 In vitro culture of erythroid precursor cells

**Figure 1:**
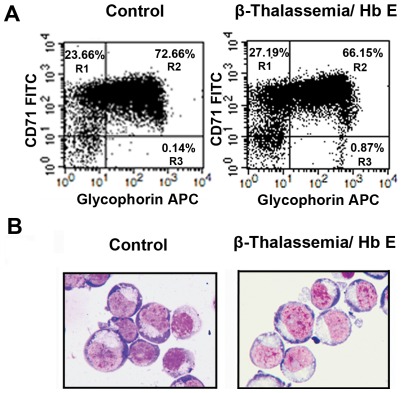
*In vitro* culture of CD34^+^-derived erythroid precursor cells. **A**) Purity of CD34^+^-derived erythroid precursor cells after cultivation for 7 days as analyzed for a control and one thalassemic patient by flow cytometry. Cells that express erythroid specific cell-surface markers, glycophorin A (CD235a) and transferrin receptor (CD71) (quadrants R1–R3), are shown as flow cytometry plot. **B**) Erythroid precursor cells were stained using Wright-Giemsa's staining. Images were captured by a microscope digital camera system (Olympus CH-2, magnification 1000×).

In this study, we have initiated a biochemical characterization of the calpain-calpastatin system in proerythroblasts derived from cultured precursor cells of thalassaemic patients chosen and selected as specified in Methods. We have cultivated samples enriched for CD34^+^ cells for 7 days under conditions promoting the differentiation into erythroid precursor cells (proerythroblasts). Purity of the cell population expanded *ex vivo* was analyzed by flow cytometric detection of the erythroid specific-cell surface markers, transferrin receptor (CD71) and glycophorin A (CD235a). More than 90% of the produced cells expressed erythroid-specific surface proteins (Fig. 1A). Differential determination of samples by Wright Giemsa's staining identified 93–95% erythroid precursor cells at the proerythroblast stage and 2–4% at the basophilic normoblast stage, whereas white blood cells comprised <1% of the samples (Fig. 1B).

### 3.2 Assay of calpain activity

**Figure 2:**
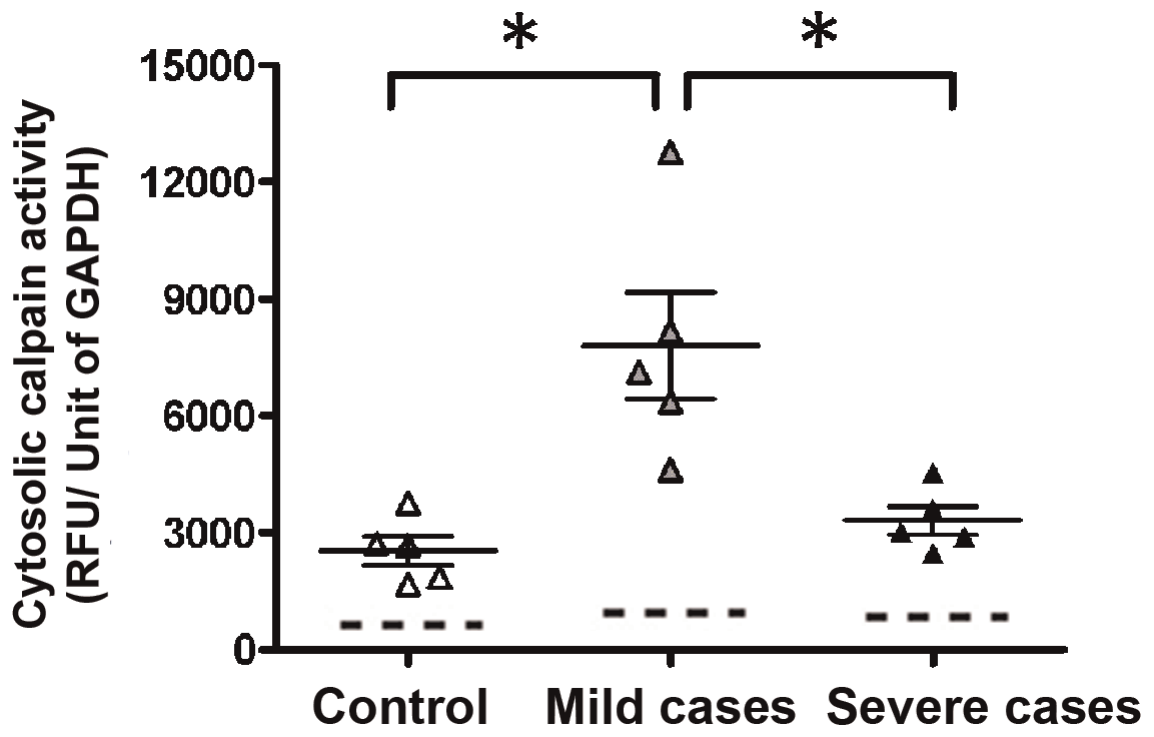
Assay of calpain activity. Analysis of cytosolic calpain activity in erythroid precursor cells of samples from controls (n = 5; n indicates number of samples analyzed), β^0^-thalassaemia/Hb E with mild clinical symptoms (n = 5), and β^0^-thalassaemia/Hb E with severe clinical symptoms (n = 5). Calpain activity is plotted as relative fluorescence units (RFU) per unit of GAPDH activity. The dashed lines represent the mean of the calpain-independent substrate hydrolysis measured in the presence of EDTA, Ca^2+^+ZLLY-fmk or calpastatin (see Methods). Data are presented as the mean ± standard error. Asterisks above the data points indicate a significance of *p*<0.05.

Calpain activity was measured in cytosolic fractions obtained from cells isolated from healthy controls and from patients with mild and severe symptoms, as described in Methods, using the fluorogenic substrate Ac-LLY-afc. The protease activity was measured also in the presence of EDTA or of the synthetic calpain inhibitor Z-LLY-fmk [20], [21] or of the protease natural inhibitor calpastatin (RNCAST300). Thus, the non-specific activity detectable in the presence of the inhibitors has been subtracted to the activity assayed in the presence of Ca^2+^ alone. Proteolysis of the fluorogenic substrate in the assay containing EDTA or calpain inhibitors was poorly detected, indicating a limited contamination by other proteases. As shown in Fig. 2, calpain activity in the 5 controls showed a mean value of approximately 2,500 RFU with a fluctuation of ±400 RFU. In the cytosol from mild cases, the mean value resulted to be increased up to 7,800 RFU with fluctuation from 5,000 to 13,000 RFU. The complete absence of superimposed values between controls and mild cases indicates that these differences are highly significant. Conversely, calpain activity in cytosol from severe cases was only 15–25% higher than levels of the control and significantly lower than in mild cases. Thus, the increase in fluorescence over the background can be attributed to the activity of calpain present in samples (Fig. 2).

### 3.3 Expression of μ-calpain

**Figure 3:**
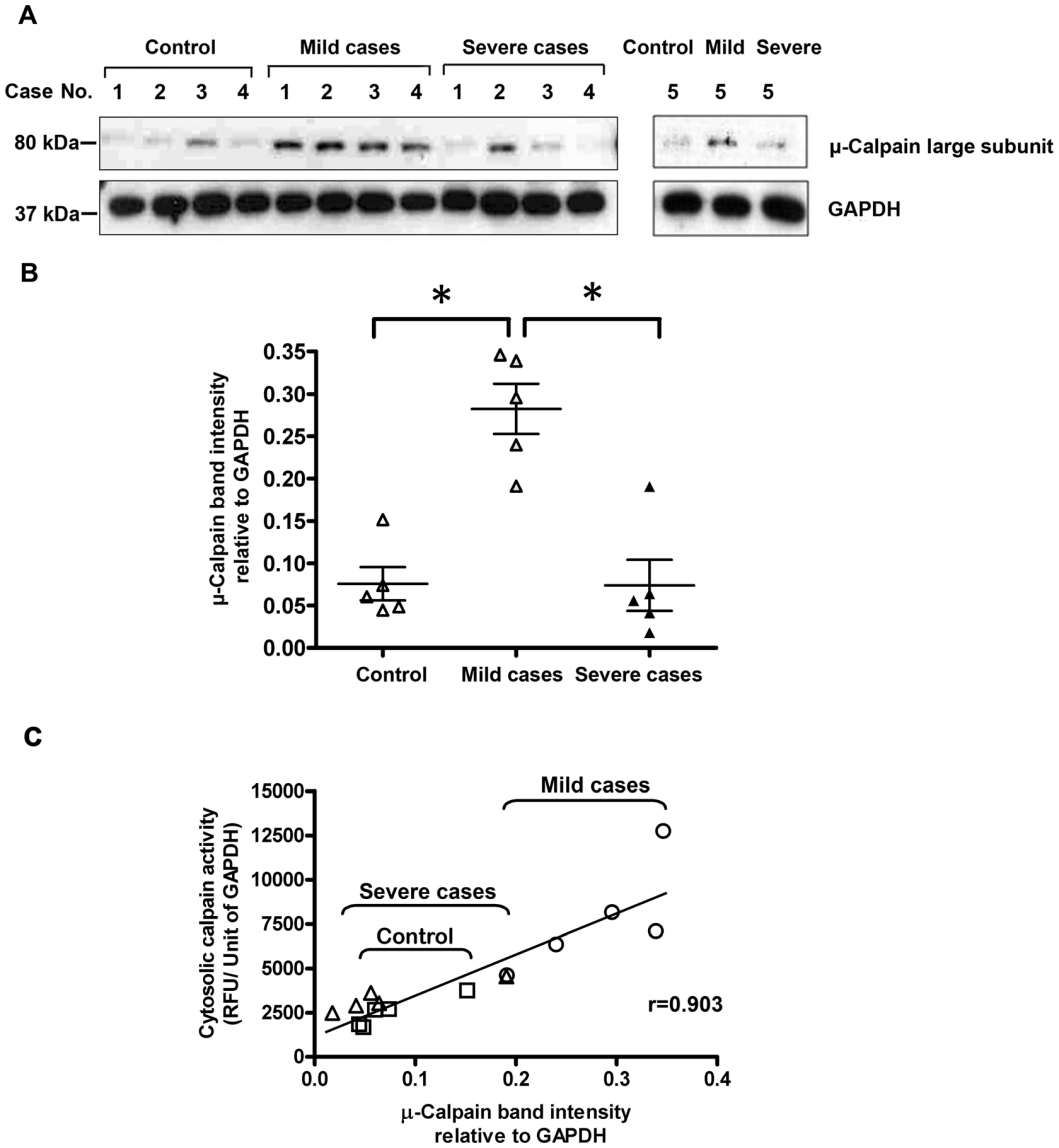
Correlation between μ-calpain protein expression and activity in individual samples of normal and β^0^-thalassaemia/Hb E proerythroblasts. **A**) Western blot analysis of μ-calpain protein expression. Immunoblots show μ-calpain present in individual samples from 5 patients. **B**) Densitometric analysis of the μ-calpain protein band. Scatter graph representing μ-calpain amounts. μ-calpain protein amounts were normalized against GAPDH band intensity. Data are presented as mean ± standard error. Asterisks above the data points indicate a significance of *p*<0.05. **C**) Correlation between μ-calpain expression and activity. Data are taken from Figure 3B and Figure 2. (□) indicates control, (**○**) patients with mild clinical symptoms, and (**Δ**) patients with severe clinical symptoms.

Since the calpain fluorimetric assays shown in Fig. 2 were not indicative of the level of calpain present in the samples, due to the presence of its natural protease inhibitor, calpastatin, we have measured the protein level of μ-calpain, the protease isoform preferentially expressed in erythrocytes [25] by immunoblotting. As shown in Fig. 3A and B, the 80 kDa protease band, corresponding to the calpain catalytic subunit, was more intense in all the 5 mild cases, as compared to controls and also to the 5 severe cases. Quantification by densitometric analysis revealed that the calpain signal detected in mild cases ranged from 2.5 to 4.5-fold significantly higher than that observed in controls and in severe cases, 3.5 to 3.7-fold higher in respect to severe cases and controls, respectively. The absence of the 78 and 75 kDa calpain bands in the immunoblots suggests that these autoproteolysed calpain forms are not accumulated in the cell and probably rapidly removed by aggregation and further digestion [25]. Thus, these data are in agreement with those showed in Fig. 2 and confirm that increased calpain activity detected in mild cases are due to a parallel increase in expression of μ-calpain. This conclusion is further supported by data showing that a direct correlation between the amounts of protein detected with the anti-calpain antibody and the calpain hydrolytic activity evaluated into the cells exists (Fig. 3C).

### 3.4 Level of calpastatin in mild and severe cases

**Figure 4:**
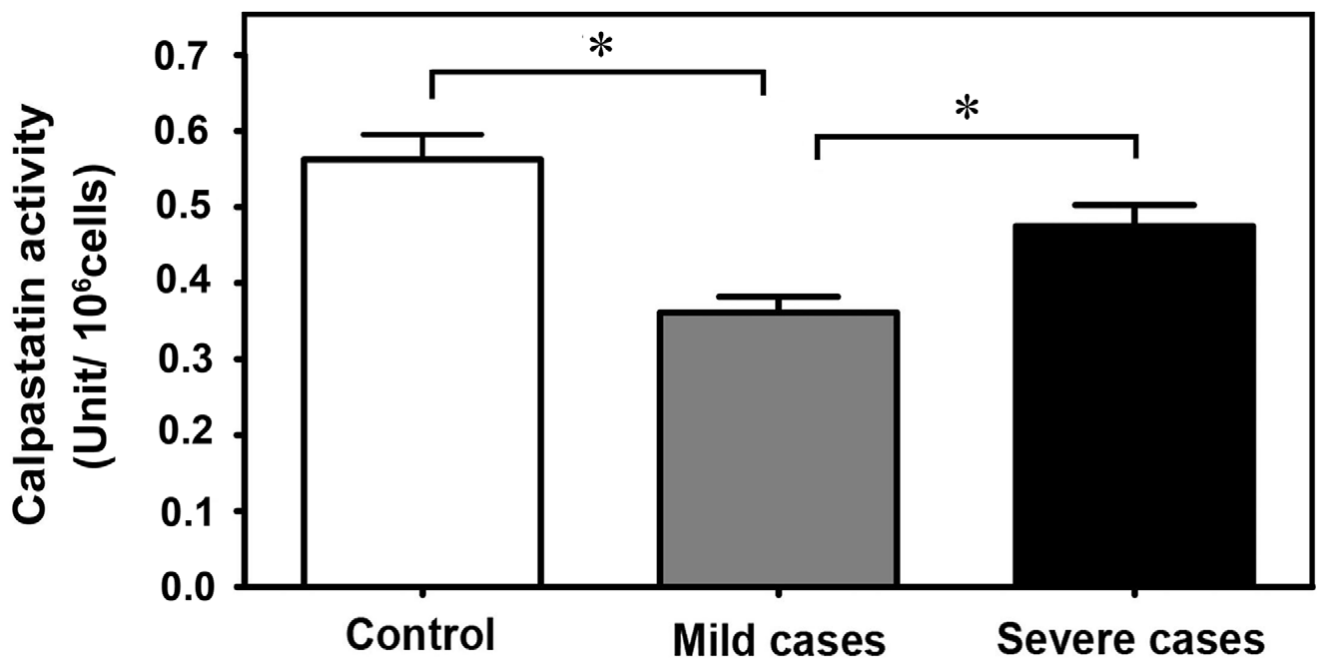
Assay of total calpastatin activity. The bar graph represents calpastatin activity in the sample groups. Calpastatin activity is shown as units of activity per 10^6^ erythroid precursor cells. Asterisks above the bars indicate a significance of *p*<0.05.

It was surprising that in cells from severe cases, the level of calpain was significantly lower compared to the mild cases. To explain this possible discrepancy, we have measured the level of calpastatin inhibitor activity in the three cell populations. As shown in Fig. 4A, calpastatin inhibitory activity is 34% reduced in cytosol from mild cases and only 16% reduced in severe cases. These observations are consistent with the level of calpain activity detected in the cytosol of these subjects and the lower availability of calpastatin present in mild cases can further enhance calpain activation.

### 3.5 Assessment of intracellular calpain activation

**Figure 5:**
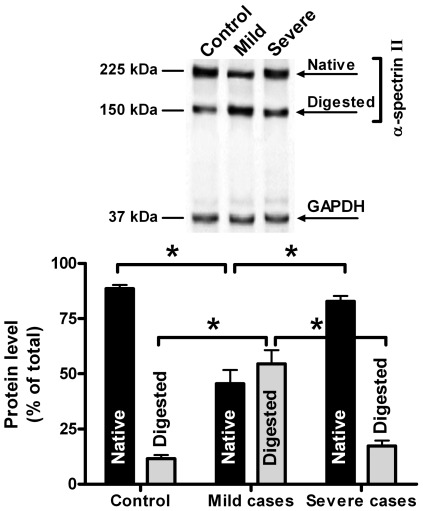
Assessment of intracellular calpain activation by spectrin degradation. Intracellular calpain activation has been evaluated by measuring the amount of the α-spectrin II proteolytic fragment. Aliquots (25 µg protein) of crude extract obtained from the three cell populations as described in Methods were submitted to 6%–10% gradient SDS-PAGE followed by Western blotting. Nitrocellulose membranes were probed by using the specific mAbs as described in Methods. Bands were quantified as in Methods. Native and digested fragment amounts of α-spectrin II were normalized against GAPDH band intensity, utilized as an internal loading control protein. The pictures in the upper panel are representative of the experiments performed. Data are presented as mean ± standard error. Asterisks above the data points indicate a significance of *p*<0.05.

Since α-spectrin II digestion by calpain has been identified in almost all Ca^2+^-loaded cells so far studied [25], [26], [27], the formation and accumulation of the low Mr calpain digested α-spectrin II generated by active calpain was evaluated by means of Western blot using the specific anti-α-spectrin II mAb. As shown in Fig. 5, the α-spectrin II fragment was detectable in all the three cell populations, although at significantly different amounts. In mild cases, the mean level of the modified spectrin resulted to be increased up to approximately 5-fold, whereas in severe cases the increase was approximately from 1.5- to 2-fold as compared to controls. The amount of the digested α-spectrin II form was thereby indicative of the extent of the different intracellular calpain activation occurring in the three cell populations. The presence of the digested α-spectrin II in control cells could be due to an involvement of the cytoskeleton digestion in normal cell function such as secretion [25], [27], [28], [29], [30]

### 3.6 Level of calpastatin in mild and severe cases

Since calpastatin can be digested by active calpain producing low molecular weight forms still retaining the inhibitory capacity corresponding to the isolated inhibitory domain [22], [24], [25], [26], [27], [31], we have then analyzed if the different calpastatin molecular species were present in mild and severe cases as compared to controls.

**Figure 6:**
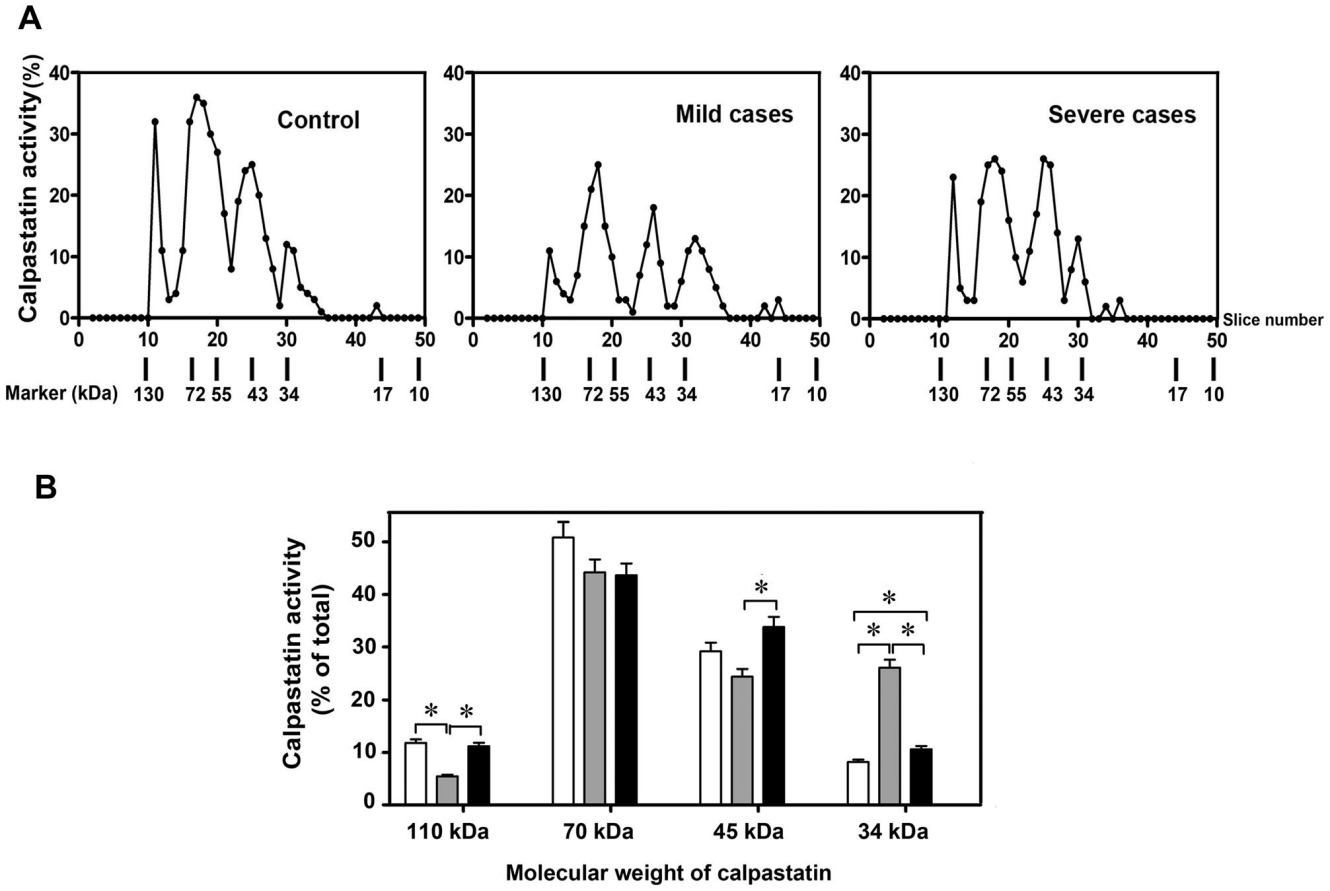
Identification of active calpastatin molecular species. **A**) Activity profiles of samples from 10^6^ erythroid precursor cells isolated from control and patients with mild and severe clinical symptoms, respectively. Numbers at the bottom refer to the molecular weight of proteins present in the gel slices. **B**) Bar graph presentation of calpastatin activity in extracted fractions of SDS-PAGE gels. Activity is presented as percentage of total calpastatin activity measured in the sample. White, grey, and black bars represent activity of calpastatin forms in samples from control, mild, and severe clinical symptoms, respectively. Asterisks above the bars indicate a significance of *p*<0.05.

As shown in Fig. 6A, in controls the predominant calpastatin species is the 70 kDa molecular form, accompanied by smaller amounts of a 45–50 kDa form. In mild cases, the two calpastatin species were largely reduced and a 34 kDa form were accumulated. These data support the hypothesis that calpain activation occurring in cells from mild cases is responsible also for the fragmentation of calpastatin promoting the appearance of significant amounts of the low molecular weight calpastatin forms. In the severe cases, the high molecular weight calpastatin forms are recovered, (Fig. 6B), thus suggesting the existence of a defence mechanism as previously identified in animal brain and muscle cells [26], [27].

## Discussion

Calpains are intracellular, neutral Ca^2+^-dependent cysteine proteases thought to be involved in numerous fundamental processes essential for cell function [25]. In humans, two isoforms (the ‘classical’ and ubiquitous μ- and m-calpain) are characterized by the 80 kDa catalytic subunit and a common regulatory 30 kDa subunit. It should be noted that only μ-calpain has been detected in human erythrocytes [32]. Previous work has demonstrated that erythrocyte μ-calpain is not only involved in the degradation of submembranous cytoskeleton proteins but also utilizes native as well as heme-deprived α- and β-globin chains as substrate [33].

Although employing a relatively limited sample format by selecting samples containing only identical mutations of β^0^-thalassaemia/Hb E disease, we have initially confirmed by a biochemical assay that calpain activity is elevated in erythroid precursor cells from patients with mild clinical symptoms. We have found that not only enzyme activity is increased but also that the amount of immunodetectable calpain protein is higher in patients with mild clinical symptoms than those of healthy controls and patients with severe clinical symptoms. Since activity of calpain is strictly regulated by its inhibitor, calpastatin, and it has been proposed that massive overactivation of calpain in erythroid precursor cells is prevented by calpastatin [16], we have investigated both calpastatin activity and calpastatin molecular forms present in our samples.

Calpastatin is a protein inhibitor with high specificity for calpain proteases [34]. It is synthesized in erythrocytes predominantly as erythrocyte-type calpastatin (70 kDa), however, both erythrocyte-type and tissue-type calpastatin (110–120 kDa) were identified in the erythroid cell line JK-1 thus suggesting that erythroid precursor cells can contain both forms of the inhibitor [35]. In general, calpastatin protein amounts are much higher than those of calpain in various cell types [36]. A balance of protein amounts between calpain and calpastatin and/or subcellular distribution of calpastatin has been anticipated as possible mechanism for the regulation of calpain activity [37], [38].

Alteration of the calpain/calpastatin ratio observed in cells from mild cases can also explain the large extent of proteolytic degradation of α-spectrin II by calpain occurring in the cell population. The recovery of calpastatin in cells from severe cases and the decrease in calpain protein are consistent with the lower degree of intracellular calpain activation in these cells.

Moreover, previous studies have demonstrated that calpastatin also serves as substrate for proteolytic degradation by calpain itself and consequently increased calpain activation has been associated with reduced calpastatin activity [26]. We have therefore decided to characterize the activity of calpastatin molecular species and its specific degradation products. By SDS-PAGE gel extraction, we detected the activity of both native of calpastatin forms, the tissue (110 kDa) and erythrocyte type (70 kDa). Moreover, calpastatin activity was observed at the lower protein molecular weights of 45 and 34 kDa. Earlier studies have suggested that enhanced formation of a 45-kDa calpastatin subunit is a result of the partial degradation of calpastatin by intracellular calpain [39]. A previous report has described the production of a 34-kDa calpastatin subunit with inhibitory activity as result of a proteolytic cleavage of the 45-kDa fragment by μ-calpain in rat liver [40]. We have observed that the activity of the 110 kDa tissue-type calpastatin was significantly reduced when compared to the 70 kDa erythrocyte-type calpastatin in proerythroblasts from patients with mild clinical symptoms. In accordance with earlier observations, it is conceivable that under conditions of elevated calpain amounts and activity, calpain-mediated proteolysis of tissue-type calpastatin occurs faster than degradation of the erythrocyte-type [35], [39]. Additionally, we observed the accumulation of the 34-kDa calpastatin fragment in patients with mild clinical symptoms, whereas amounts of this fragment in the control samples were almost negligible and slightly increased in patients with severe clinical symptoms. As total calpastatin activity was significantly reduced in samples from mild cases, it is therefore conceivable that calpain-mediated degradation of calpastatin generates the active 34-kDa fragment and smaller inactive peptides as described earlier [26].

Finally, the observed decrease in both calpain activity and protein can be due to a higher rate of activation/inactivation process at which calpain undergoes, causing a consumption of the protease. This hypothesis is also supported by the higher extent of α-spectrin II degradation in cells in which also higher calpain activity was detectable. The presence of high amounts of low molecular mass calpastatin forms, produced also by active calpain [22], [26], [27] is also indicative of calpain activation, whereas the recovery in the inhibitor level in cells from severe cases is consistent with a defence mechanism shown to operate under conditions of prolonged alteration of Ca^2+^ – homeostasis [22], [27], [31].

Taken together, our results would suggest that increased calpain protein expression under elevated intracellular calcium concentration in thalassemic proerythroblasts [41] may lead to increased calpain activity and enhances the degradation of calpastatin in patients with mild clinical symptoms. Elevated calpain activity in patients with mild clinical symptoms has been previously suggested to contribute to an accelerated degradation of its committed substrate, free α-globin chains, resulting in reduced precipitation of globin, a lower degree of ineffective erythropoiesis and amelioration of the pathophysiological symptoms of β-thalassaemia/Hb E [9], [13]. However, further investigations are required to address the mechanism by which calpain protein expression becomes elevated in mild forms of β-thalassaemia/Hb E, whereas it seems retaining a basal level in severe forms of β-thalassaemia/Hb E. It can not be ruled out at present that additional proteolytic functions contribute to the modification of disease severity [32], [42]. It is also important to note that a multitude of factors including genetic disposition and their possible interaction determining the phenotypic variability in the clinical manifestations and physiological response to genetic defects in thalassaemic diseases remains largely to be investigated [43]. Nevertheless, this study has demonstrated for the first time directly a possible association between disease severity and calpain activation in thalassaemia.

## Supporting Information

File S1 **Approval document of ethical clearance for the research work described herein by the committee on human rights related to human experimentation of Mahidol University, Bangkok, Thailand.**
(JPG)

File S2 **Informed consent form signed by the patients participating in this study.** Patients have signed this form translated into their native (Thai) language.
(DOC)
