# Supplementary figures and images for: Clinical Severity of β-thalassaemia/Hb E Disease Is Associated with Differential Activities of the Calpain-Calpastatin Proteolytic System
Source: PLoS One. 2012 May 16;7(5):e37133. doi: 10.1371/journal.pone.0037133 (PMC3353910; doi:10.1371/journal.pone.0037133)

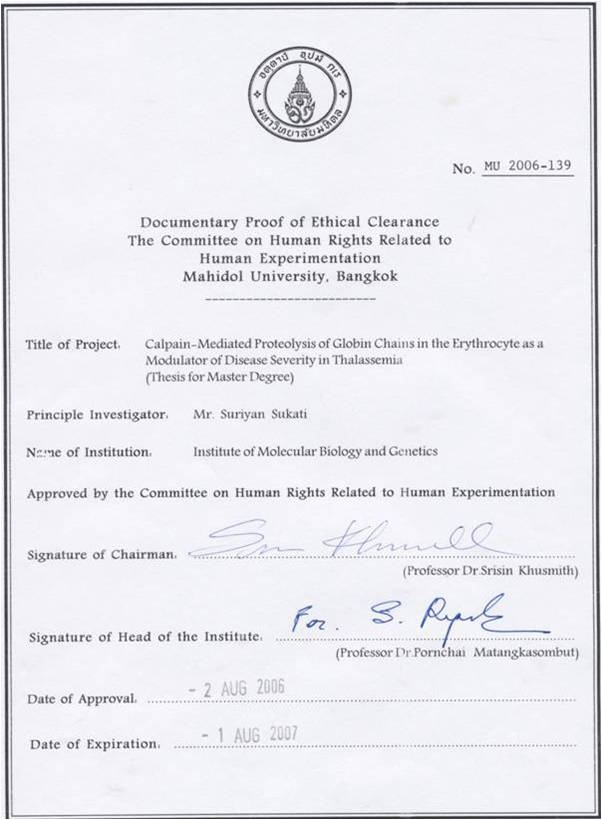

Supplement: File S1 — Approval document of ethical clearance for the research work described herein by the committee on human rights related to human experimentation of Mahidol University, Bangkok, Thailand. (JPG) [file pone.0037133.s001.jpg]
