# Supplementary material for: Clinical Severity of β-thalassaemia/Hb E Disease Is Associated with Differential Activities of the Calpain-Calpastatin Proteolytic System
Source: PLoS One. 2012 May 16;7(5):e37133. doi: 10.1371/journal.pone.0037133 (PMC3353910; doi:10.1371/journal.pone.0037133)
Supplement: File S2 — Informed consent form signed by the patients participating in this study. Patients have signed this form translated into their native (Thai) language. (DOC) [file pone.0037133.s002.doc]

**Informed consent form on human rights related to experimentation**

**Title:** Calpain-mediated proteolysis of globin chains in the erythrocyte as a modulator of disease severity in thalassemia.

Date for consent, Date…………………..Month……………………Year

Before I have signed the informed consent form of this research, I have received an explanation of the research objective, methods, risks, and side effects caused by research or taking drug from researcher, including the advantages in detail of this research and I well understand.

Researcher guarantees to answer the questions for any doubts without [concealment](http://dict.longdo.com/search/concealment).

I am free to withdraw at any time from the project without my medical care affected.

Researcher warrants that my identity will be kept confidential and not be revealed in any information, but will be presented only in term of the research conclusion involving academic knowledge to related institutes.

Researcher warrants that if I get any dangerous from research, I will receive medical care without payment, [recompense](http://dict.longdo.com/search/recompense) during medical care and disability which probably occur.

Researcher warrants additional information that effect to the study will be informed me without [concealment](http://dict.longdo.com/search/concealment).

I have read this consent and been given freely sign in this informed consent form.

Signature……………………………………………………………………...... Participant

Signature……………………………………………………………………….. Witness

Signature……………………………………………………………………….. Witness

In case of [illiteracy](http://dict.longdo.com/search/illiteracy), participant must have consciousness and designate that I cannot read, but researcher read them to me until understanding and then I have signed or thumb fingerprints of right hand.

Signature………………………………………………………………………. Participant

Signature……………………………………………………………………….. Witness

Signature……………………………………………………………………….. Witness

In case of less than 18 years of age, the informed consent must be obtain from parents or legal guardian.

Signature……………………………………………………………………Parents or

legal guardian.

Signature……………………………………………………………………….. Witness

Signature……………………………………………………………………….. Witness

In case of [wobbliness](http://dict.longdo.com/search/wobbliness), for example, unconscious, legal legally delegate, parents or relatives, sign for giving the consent.

Signature………………………………………………………………….. Legally delegate /parents/relatives

Signature……………………………………………………………………….. Witness

Signature……………………………………………………………………….. Witness
